# Concurrent Alterations in Selected mRNAs and miR-30 Family Members in Canine Osteoarthritic Joint Tissues

**DOI:** 10.3390/biomedicines14092024

**Published:** 2026-09-09

**Authors:** Gabriella Guelfi, Vicente F. Ratto, Camilla Capaccia, Francesco Ciancabilla, David Forti, Leonardo Leonardi, Antonello Bufalari, Arcangelo Liso, Margherita Maranesi

**Affiliations:** 1 Department of Veterinary Medicine, University of Perugia, 06126 Perugia, Italy; vicentefrancisco.rattovalderrama@dottorandi.unipg.it (V.F.R.); camilla.capaccia@dottorandi.unipg.it (C.C.); francesco.ciancabilla@studenti.unipg.it (F.C.); david.forti@dottorandi.unipg.it (D.F.); leonardo.leonardi@unipg.it (L.L.); antonello.bufalari@unipg.it (A.B.); margherita.maranesi@unipg.it (M.M.); 2 Department of Medicine and Surgery, University of Perugia, 06132 Perugia, Italy; arcangelo.liso@unipg.it

**Keywords:** osteoarthritis, miR-30 family, post-transcriptional regulation, joint capsule, synovial membrane, canine, RT-qPCR

## Abstract

**Background:** Osteoarthritis (OA) is a chronic whole-joint disease involving extracellular matrix remodeling, inflammatory signaling, immune–stromal interactions, and sensory-associated processes. MicroRNAs (miRNAs) may contribute to post-transcriptional regulation within these interconnected molecular domains, but concurrent mRNA and miRNA expression patterns remain insufficiently characterized in naturally occurring canine OA. **Methods:** A total of 12 formalin-fixed, paraffin-embedded (FFPE) canine tissue specimens were classified as osteoarthritic joint tissues (OA; n = 7) and control joint tissues without radiographic or histopathological evidence of OA (C-JT; n = 5). RT-qPCR was used to quantify 17 selected mRNAs involved in extracellular matrix remodeling, chronic inflammatory signaling, immune–stromal interactions, and neuroinflammatory and sensory-associated processes, together with five miR-30 family members. Statistical comparisons were performed on ΔCq values using two-sided unpaired Welch’s *t*-tests followed by Benjamini–Hochberg false discovery rate correction. **Results:** Compared with C-JT, OA tissues showed significantly higher expression of ADAMTS5, MMP2, MMP13, COL2A1, SPARC, HP, VEGFA, IGFBP6, and ASIC3 after false discovery rate correction. MMP9, IL1B, IL1R1, IL1RAP, JAK2, TYK2, NGFB, and NTRK1 did not differ significantly between the two joint-tissue groups. MiR-30b was significantly downregulated in OA, whereas miR-30c, miR-30d, and miR-30e were significantly upregulated; miR-30a did not differ significantly. Exploratory database-supported miRNA–gene connectivity was used for candidate prioritization but was not interpreted as evidence of direct regulation in canine joint tissue. **Conclusions:** Canine osteoarthritic capsulosynovial tissues exhibit concurrent alterations in selected mRNAs and miR-30 family members across matrix-remodeling, immune–stromal, and sensory-associated molecular domains. These findings identify OA-associated expression patterns but do not establish pathway activation, direct miRNA–mRNA regulation, or disease progression. They provide a focused molecular basis for future mechanistic studies of post-transcriptional regulation in naturally occurring canine OA.

## 1. Introduction

Osteoarthritis (OA) is increasingly recognized as a disease with a prolonged preclinical phase, during which molecular and structural alterations may precede overt clinical manifestations [1]. The frequent discordance between structural joint damage and symptom severity contributes to the marked clinical and biological heterogeneity of OA [2,3,4,5]. During this clinically silent but biologically active phase, conventional diagnostic approaches may show limited sensitivity in detecting early joint alterations [6]. Such heterogeneity reflects the involvement of multiple biological processes operating across different levels of molecular regulation [7].

At the molecular level, OA is a whole-joint disorder involving interconnected processes related to extracellular matrix remodeling, chronic inflammatory signaling, immune–stromal interactions, and neuroinflammatory and sensory-associated pathways [8]. Accordingly, the targeted panel included transcripts spanning these domains, as well as matrix-remodeling transcripts such as ADAMTS5, MMP2, MMP13, and COL2A1, reflecting proteolytic and structural components of extracellular matrix turnover [9]; immune–stromal and tissue-response transcripts such as SPARC, VEGFA, IGFBP6, and HP, relevant to remodeling of the synovial microenvironment [10]; and ASIC3 as a sensory-associated transcript implicated in arthritis-related nociceptive mechanisms [11]. However, changes in transcript abundance within these domains should not, by themselves, be interpreted as evidence of pathway activation, protein activity, matrix degradation, or pain severity. Because these processes are interconnected, OA cannot be understood solely through isolated transcriptional alterations; epigenetic and post-transcriptional mechanisms may also contribute to shaping the molecular profile of affected joint tissues [12].

In this context, microRNAs (miRNAs) represent a relevant post-transcriptional regulatory layer because of their potential to influence multiple transcripts within interconnected molecular networks. miRNAs have been implicated in extracellular matrix homeostasis, inflammatory signaling, cellular differentiation, and other biological processes relevant to OA [13]. Nevertheless, concurrent alterations in miRNA and mRNA abundance indicate potential regulatory relationships but do not, by themselves, demonstrate direct target regulation or compensatory activity. Among the miRNA families implicated in musculoskeletal biology, the miR-30 family has been associated with extracellular matrix regulation, inflammatory responses, cellular differentiation, and bone and joint remodeling [14,15]. In human OA cartilage and chondrocytes, miR-30a has been implicated in the regulation of ADAMTS5 through the IL-1β/AP-1/miR-30a axis [16]. MiR-30b-5p has also been associated with inflammatory and cartilage-related responses involving the SIRT1–FOXO3A–NLRP3 axis [16], whereas a broader review has highlighted potentially distinct and context-dependent functions of individual miR-30 family members in bone and joint diseases [15]. However, the concurrent expression of these miRNAs in canine OA joint tissues and their potential relationships with selected OA-associated transcripts remain insufficiently characterized.

The dog represents a relevant spontaneous model of OA because the disease develops naturally in anatomically and biomechanically complex joints and shares several clinical, structural, pathological, environmental, and lifestyle-related features with human OA [17]. Naturally occurring canine OA can therefore complement experimentally induced and rodent models by reflecting aspects of the chronic and multifactorial nature of the disease. The translational relevance of the canine model is further supported by substantial genomic conservation between dogs and humans [18,19]. The present study investigated concurrent mRNA and miRNA expression patterns in canine osteoarthritic joint capsule specimens containing synovial membrane and in anatomically comparable control joint tissues without radiographic or histopathological evidence of OA. The analysis compared OA and control joint tissues, while enrichment analyses were used to characterize the biological processes represented within the targeted gene panel. An exploratory miRNA–gene network analysis was used to prioritize miR-30 family members showing database-supported connectivity with selected transcripts potentially relevant to joint homeostasis and remodeling. Overall, the study aimed to characterize concurrent OA-associated alterations in selected mRNAs and miR-30 family members and to provide a focused molecular framework for future mechanistic studies of post-transcriptional processes involved in joint homeostasis and remodeling responses.

## 2. Materials and Methods

### 2.1. Case Selection and Inclusion Criteria

The present retrospective study included 12 formalin-fixed, paraffin-embedded (FFPE) canine tissue specimens retrieved from the archives of the Veterinary Pathology Section and the Veterinary Teaching Hospital, Department of Veterinary Medicine, University of Perugia, covering the period 2018–2025. Specimens originated from surgical biopsies or necropsies and were selected based on the available clinical, radiographic, and histopathological records, adequate tissue preservation, and suitability for molecular analysis. Available clinical records were reviewed to exclude documented systemic inflammatory or infectious diseases, and additional exclusion criteria are detailed in Supplementary Material S1. Each specimen was obtained from a different dog; therefore, all samples represented biologically independent animals, and no matched or contralateral specimens were included. The specimens were assigned to two predefined tissue categories: seven osteoarthritic joint tissues (OA)and five control joint tissues without radiographic or histopathological evidence of OA (C-JT). All OA and C-JT specimens consisted of joint capsule with synovial membrane collected from the knee or elbow. The FFPE sections used for RNA extraction were cut adjacent to the H&E-stained sections used for histopathological classification, thereby improving block-level correspondence between the histological and molecular analyses without implying cellular colocalization. The extracted sections comprised the capsulosynovial tissue compartment and did not include articular cartilage. Inclusion in the OA group required concordant radiographic and histopathological evidence of established OA according to the predefined criteria reported in Supplementary Material S1. All OA specimens fulfilled the criteria corresponding to radiographic Grades 2–3 [20]. Inclusion in the C-JT group required a Grade 0 radiographic classification, defined by the absence of radiographic evidence of OA, including the absence of osteophytes, normal subchondral bone, and preserved joint contours, together with no histopathological evidence of OA in the examined joint tissue. Grade 1 joints were excluded to provide a clear comparison between radiographically normal Grade 0 joints and established OA, thereby reducing the risk of classifying borderline or incipient alterations as either normal or established disease. Consequently, the present findings apply to established OA and should not be generalized to early-stage disease. Tissues collected at necropsy were fixed in 10% neutral-buffered formalin and processed according to the established routine diagnostic histology procedures of the Veterinary Pathology Section. Archived surgical biopsy specimens had undergone the same processing workflow. Because of the retrospective nature of the study, fixation duration, postmortem interval, sample-specific collection year, surgical versus necropsy origin, and complete processing records were not consistently available for all specimens. Consequently, the distribution of these preanalytical variables could not be reliably compared between the OA and C-JT groups. This limitation was considered when interpreting the molecular findings. Histopathological evaluation was performed on 4 μm sections stained with hematoxylin and eosin (H&E). The sections were examined by L.L., a board-certified veterinary pathologist, using light microscopy to confirm tissue identity, preservation, and diagnostic classification. Breed, age, sex, sampling site, Sample ID, and Animal ID are summarized in Table 1a, whereas radiographic grade and histopathological classification are reported in Table 1b.

### 2.2. Experimental Design

**Figure 1:**
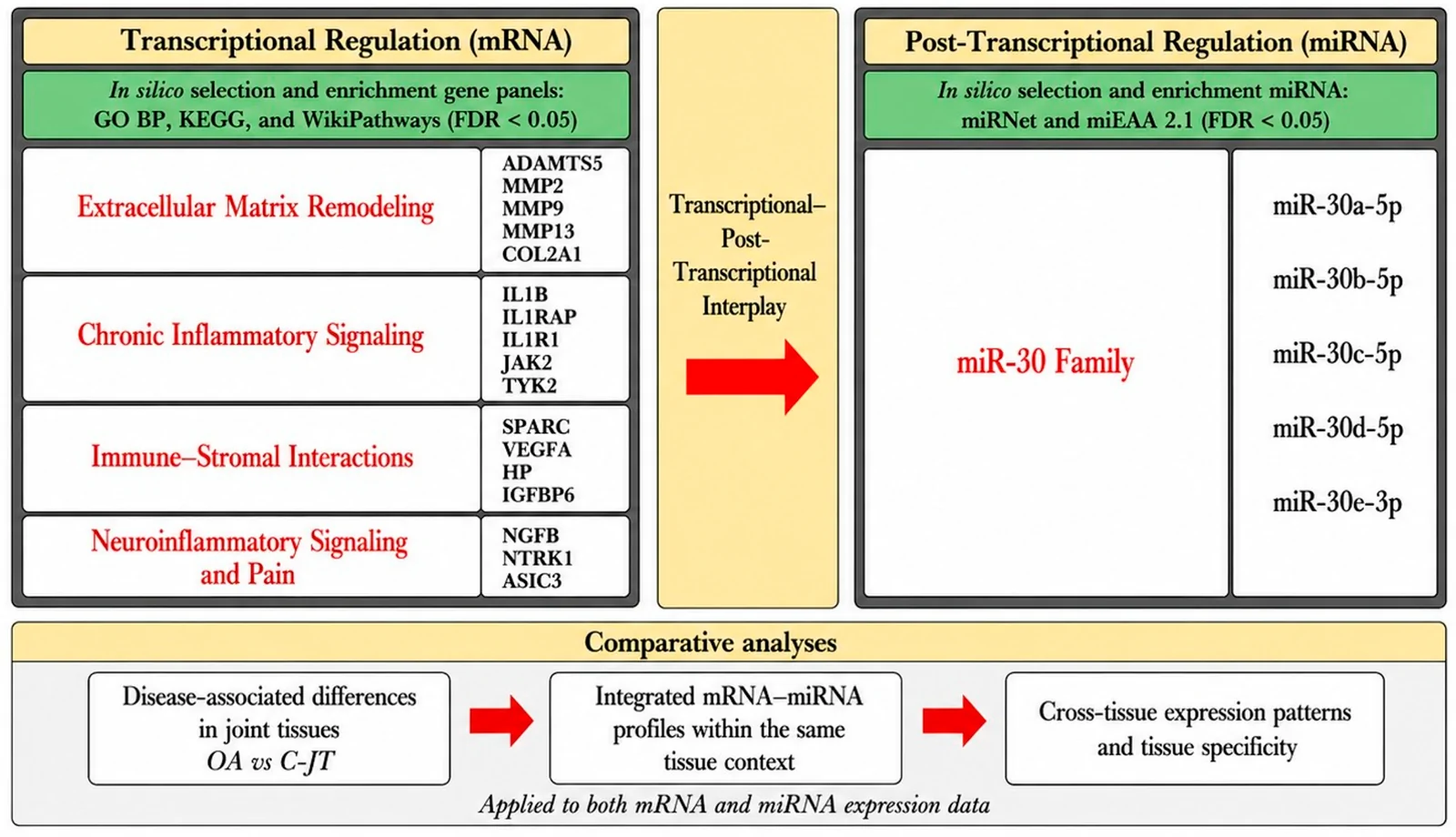
Experimental design and analytical workflow. Seven osteoarthritic joint tissues (OA) and five control joint tissues (C-JT) were included in the study. All specimens were obtained from biologically independent dogs, and no matched or contralateral specimens were included. Concurrent mRNA and miRNA profiling was performed on adjacent FFPE sections to improve block-level correspondence between the transcriptional and post-transcriptional measurements, without implying cellular colocalization. The (**left panel**) summarizes the selected mRNA targets grouped into four functional domains: extracellular matrix remodeling, chronic inflammatory signaling, immune–stromal interactions, and neuroinflammatory and sensory-associated processes. The (**right panel**) shows the miR-30 family selected for targeted expression analysis based on its connectivity with the predefined gene panel. Comparative analyses included OA versus C-JT comparison applied to mRNA and miRNA expression data. The cross-tissue comparison was considered exploratory and was not used to establish OA specificity or direct miRNA–mRNA regulation.

OA-associated molecular differences were investigated using parallel targeted mRNA and miRNA profiling of FFPE tissue specimens. For each specimen, molecular analyses were performed on sections adjacent to those used for histopathological classification, thereby improving block-level correspondence without implying cellular colocalization. The study included seven osteoarthritic joint tissues (OA) and five control joint tissues (C-JT), all obtained from biologically independent dogs. The analytical workflow comprised two comparisons. The analysis compared OA and C-JT specimens to identify OA-associated differences in mRNA and miRNA abundance in anatomically comparable joint tissues. The targeted mRNA panel was organized into four predefined functional domains: extracellular matrix remodeling, chronic inflammatory signaling, immune–stromal interactions, and neuroinflammatory and sensory-associated processes. Because the genes had been selected in advance to represent these domains, enrichment analysis was used to annotate the biological composition of the panel rather than to identify previously unknown OA pathways. Candidate miRNAs were prioritized through an exploratory miRNA–gene network analysis performed in miRNet using interactions catalogued in miRTarBase. The miR-30 family was selected for targeted expression analysis because its members showed database-supported connectivity with multiple transcripts across the predefined functional domains. Database-supported miRNA–target interactions were considered hypothesis-generating and were not interpreted as evidence of direct regulation, compensatory activity, or a central regulatory role in the canine tissues analyzed. Accordingly, mRNA and miRNA expression data were examined as concurrent but distinct molecular readouts, without inferring direct miRNA–mRNA regulation or pathway activation. The overall experimental design and analytical workflow are summarized in Figure 1.

### 2.3. Selection and Functional Annotation of Candidate mRNAs and Network-Based Prioritization of Candidate miRNAs

Candidate target genes were selected through a targeted literature-based approach and were subsequently subjected to functional annotation. A targeted literature search was conducted in PubMed and Scopus between January 2025 and January 2026, including records available up to January 2026. Separate searches combined the terms “osteoarthritis” OR “OA” with the species terms “canine” OR “dog,” the tissue terms “synovium,” “synovial membrane,” or “joint capsule,” and terms related to extracellular matrix remodeling, inflammation, immune–stromal interactions, and neuroinflammation. The search was targeted rather than systematic and was performed to support the a priori selection of a focused RT-qPCR panel. Genes were included in the final panel when at least two independent studies supported their OA-related differential expression or biological involvement. Functional annotation of the selected gene panel was performed using the Enrichr platform [21], accessed in January 2026 [22,23], querying Gene Ontology Biological Process [24], KEGG [25], WikiPathways 2024 Human [26], and BioPlanet. Enrichment significance was evaluated using Benjamini–Hochberg-adjusted *p*-values, with a false discovery rate threshold of FDR < 0.05. Statistically significant terms retained for reporting, together with their database identifiers, overlapping genes, and Benjamini–Hochberg-adjusted *p*-values, are provided in Supplementary Tables S1a–d. No custom background gene set was specified, and the default background implemented for each Enrichr library was used. Because the genes had been selected in advance to represent OA-related processes, enrichment analysis was used to annotate the biological composition of the panel rather than to identify previously unknown OA pathways. The selected genes were organized into four predefined functional domains: extracellular matrix remodeling, chronic inflammatory signaling, immune–stromal interactions, and neuroinflammatory and sensory-associated processes. This classification was used solely to organize the targeted panel and was not considered independent evidence of the involvement of these genes in OA. Candidate miRNAs were prioritized through an exploratory miRNA–gene network analysis performed in miRNet, accessed in August 2026 [27], using interactions catalogued in miRTarBase as experimentally supported in previously reported experimental systems [28]. For the initial candidate ranking, miRNAs connected with at least two genes within the selected panel were retained. The miR-30 family was selected for targeted expression analysis because four of the five experimentally measured mature strands met this criterion and showed database-supported connectivity with transcripts belonging to the predefined functional domains. miR-30b-5p, which showed a single connection, was retained as part of the predefined miR-30 family panel evaluated experimentally. Following verification of assay identity, the final network interpretation was restricted to the exact mature miRNA strands measured experimentally, including miR-30e-3p (MIMAT0000693; CUUUCAGUCGGAUGUUUACAGC). This network analysis was used solely for candidate prioritization. Database-supported miRNA–target interactions were considered hypothesis-generating and were not interpreted as evidence of direct miRNA–mRNA regulation, compensatory activity, or a central regulatory role for the miR-30 family in canine OA.

### 2.4. Total RNA Isolation and Quantification

Total RNA, including miRNAs, was extracted from FFPE tissue samples using the miRNeasy FFPE Kit (QIAGEN, Hilden, Germany), according to the manufacturer’s instructions. For each specimen, three 5 μm-thick FFPE tissue sections were used for RNA extraction. FFPE tissue sections were deparaffinized using xylene followed by graded ethanol washes to reduce residual paraffin. Samples were then incubated at 56 °C in the kit-supplied lysis buffer containing proteinase K to release RNA from the tissue sections. The resulting lysate was treated with DNase to remove residual genomic DNA. The kit-supplied binding buffer and ethanol were subsequently added to establish the conditions required for RNA binding to the silica membrane of the spin column. Total RNA was bound to the membrane, while impurities were removed during the subsequent washing steps. RNA was eluted twice from the same spin-column membrane, each time using 20 μL of RNase-free water. The two eluates were retained separately and quantified independently, and the suitability of the second eluate for downstream analysis was evaluated according to whether its RNA concentration was sufficient to provide the required input amount. According to the manufacturer’s protocol, 1 μL of the UniSp2/UniSp4 RNA Spike-in Mix (QIAGEN, Aarhus, Denmark) was added to the lysis buffer before RNA isolation to monitor technical extraction performance. The spike-in controls were not interpreted as measures of endogenous miRNA integrity or biological abundance. RNA concentration and absorbance-based purity ratios were assessed by spectrophotometry using a NanoDrop™ 2000/2000c instrument (Thermo Fisher Scientific, Waltham, MA, USA), whereas total RNA concentration was independently quantified by fluorometry using the Qubit™ RNA Assay Kit (Life Technologies, Carlsbad, CA, USA). Small-RNA concentration was additionally estimated using the Qubit™ microRNA Assay Kit (Thermo Fisher Scientific, Waltham, MA, USA). RNA quality was evaluated using the Qubit™ RNA IQ Assay Kit (Thermo Fisher Scientific, Waltham, MA, USA). RNA IQ values were interpreted as assay-specific indicators of RNA quality and were not considered equivalent to RNA integrity numbers obtained by electrophoretic methods. RNA samples were stored at −80 °C until further analysis.

### 2.5. Gene Expression Profiling Through RT-qPCR

For each sample, 40 ng of total RNA was used as input for reverse transcription in a final reaction volume of 20 μL using the iScript™ cDNA Synthesis Kit (Bio-Rad, Hercules, CA, USA), according to the manufacturer’s instructions. No-reverse-transcriptase controls were processed in parallel to assess potential genomic DNA contamination. The resulting cDNA was diluted 1:10. A 3 μL aliquot of diluted cDNA was then subjected to target-specific preamplification in a final volume of 20 μL containing 10 μL of SsoAdvanced™ PreAmp Supermix (Bio-Rad, Hercules, CA, USA), 1 μL of a pooled mixture containing the TaqMan Gene Expression Assays listed in Table 2, and 6 μL of nuclease-free water. Preamplification was performed with an initial activation step at 95 °C for 3 min, followed by 10 cycles of denaturation at 95 °C for 15 s and annealing/extension at 58 °C for 4 min. The same assay pool and preamplification conditions were applied to all samples. qPCR was subsequently performed in a final volume of 20 μL containing 10 μL of SsoAdvanced™ Universal Probes Supermix (Bio-Rad, Hercules, CA, USA), 1 μL of the preamplified product, 1 μL of the individual TaqMan Gene Expression Assay corresponding to each target (Table 2), and 8 μL of nuclease-free water. qPCR cycling consisted of an initial activation step at 95 °C for 30 s, followed by 40 cycles of denaturation at 95 °C for 5 s and annealing/extension at 60 °C for 5 s. Both preamplification and qPCR were carried out using a StepOnePlus™ Real-Time PCR System (Applied Biosystems, Carlsbad, CA, USA). Amplification curves were visually inspected, and quantification cycle (Cq) values were determined using StepOne Software v2.3 (Applied Biosystems, Carlsbad, CA, USA).

### 2.6. miRNA Expression Profiling Through RT-qPCR

cDNA synthesis was performed using the miRCURY LNA RT Kit (QIAGEN, Hilden, Germany), according to the manufacturer’s instructions. Each 10 µL reaction contained 2 µL of 5× miRCURY RT Reaction Buffer, 1 µL of 10× miRCURY RT Enzyme Mix, 0.5 µL of synthetic UniSp6 spike-in RNA, 10 ng of total RNA, and RNase-free water to a final volume of 10 µL. The UniSp6 spike-in was included to monitor reverse-transcription performance and detect substantial technical inhibition; it was not used for normalization of endogenous miRNA expression. Reactions were incubated at 42 °C for 60 min, followed by enzyme inactivation at 95 °C for 5 min. qPCR was performed using the miRCURY LNA SYBR Green PCR Kit (QIAGEN, Hilden, Germany) in a final reaction volume of 10 µL containing 5 µL of SYBR Green Master Mix, 3 µL of cDNA diluted 1:60, 1 µL of the miRNA-specific miRCURY LNA PCR primer assay listed in Table 3, and 1 µL of RNase-free water.

qPCR cycling was performed as described [29]. Amplification was performed using a StepOnePlus™ Real-Time PCR System (Applied Biosystems, Carlsbad, CA, USA). UniSp2 and UniSp4 were quantified to assess the consistency of the RNA-extraction procedure, whereas UniSp6 was quantified to monitor reverse-transcription and amplification performance. The UniSp2/UniSp4 RNA Spike-in Mix was formulated such that UniSp2 was present at a concentration 100-fold higher than UniSp4. The expected difference between their Cq values was used as a technical quality-control indicator and was not interpreted as evidence of endogenous miRNA integrity. Each biological sample was analyzed in three technical replicates. Only reactions showing a Cq ≤ 35 and a single melting-curve peak were retained for analysis. Technical replicates showing inconsistent amplification curves were individually inspected before the mean Cq value was calculated from the accepted replicates using StepOne Software v2.3 (Applied Biosystems, Carlsbad, CA, USA).

### 2.7. Endogenous Reference Selection: mRNA and miRNA

The expression stability of three candidate reference genes (ACTB, GAPDH, and RPS18) and three candidate reference miRNAs (miR-26a-5p, miR-103a-3p, and miR-186-5p) was evaluated using four established algorithms: Delta Ct [31], BestKeeper [32], NormFinder [33], and GeNorm [34]. The stability values assigned to each candidate by each algorithm were then integrated using RefFinder to generate a comprehensive stability score [35]. Relative expression values were calculated by the 2^^−^ΔCq method [36], with normalization against the most stable endogenous control (EC) gene (ΔCq = Cq_target gene − Cq_best EC gene) or miRNA (ΔCq = Cq_target miRNA − Cq_best EC miRNA).

### 2.8. Statistical Analysis

Gene and miRNA expression analyses were performed using ΔCq values, calculated for each specimen as the mean Cq value of the target across accepted technical replicates minus the mean Cq value of the corresponding endogenous reference. Technical replicates were averaged before statistical analysis, and each tissue specimen was treated as one biological observation. Statistical inference was performed exclusively on ΔCq values for the primary comparison between osteoarthritic joint tissues (OA; n = 7) and control joint tissues (C-JT; n = 5). The distribution of ΔCq values was assessed separately for each target and group by visual inspection of individual-value plots and using the Shapiro–Wilk test. As no marked deviations from normality were identified, between-group comparisons were performed using two-sided unpaired Welch’s *t*-tests. Raw *p*-values were adjusted using the Benjamini–Hochberg false discovery rate procedure independently across the seventeen mRNA targets and the five miRNA targets. Statistical significance was determined based on FDR-adjusted *p*-values, with FDR < 0.05 considered statistically significant. All targets were evaluated in the complete set of biological specimens available for the primary comparison (OA, n = 7; C-JT, n = 5). For this comparison, the estimated difference in mean ΔCq values with its 95% confidence interval, the raw *p*-value, and the FDR-adjusted *p*-value were reported. Fold changes were calculated using the 2^−ΔΔCq method, with C-JT used as the calibrator. Statistical analyses and graphical outputs were generated using GraphPad Prism version 8 (GraphPad Software, San Diego, CA, USA).

## 3. Results

### 3.1. Histopathological Characterization of OA and Control Tissues

Available macroscopic descriptions of the OA joints indicated irregular articular surfaces, focal cartilage erosion, and varying degrees of joint capsule thickening, whereas control joint specimens were described as having smooth articular surfaces without evident macroscopic abnormalities. Histological evaluation of H&E-stained sections showed OA-associated morphological changes, including synovial lining hyperplasia, increased synovial cellularity, and inflammatory cell infiltration. In several OA specimens, loss of tissue organization and architectural disruption were also observed. In contrast, control joint tissues showed preserved synovial architecture and no evident OA-associated inflammatory or structural alterations. All seven OA joint specimens fulfilled the predefined radiographic and histopathological criteria for established OA, whereas all five control joint tissues were classified as radiographic Grade 0 and showed no histopathological evidence of OA.

### 3.2. Functional Annotation of the Selected mRNA Target Panel

**Figure 2:**
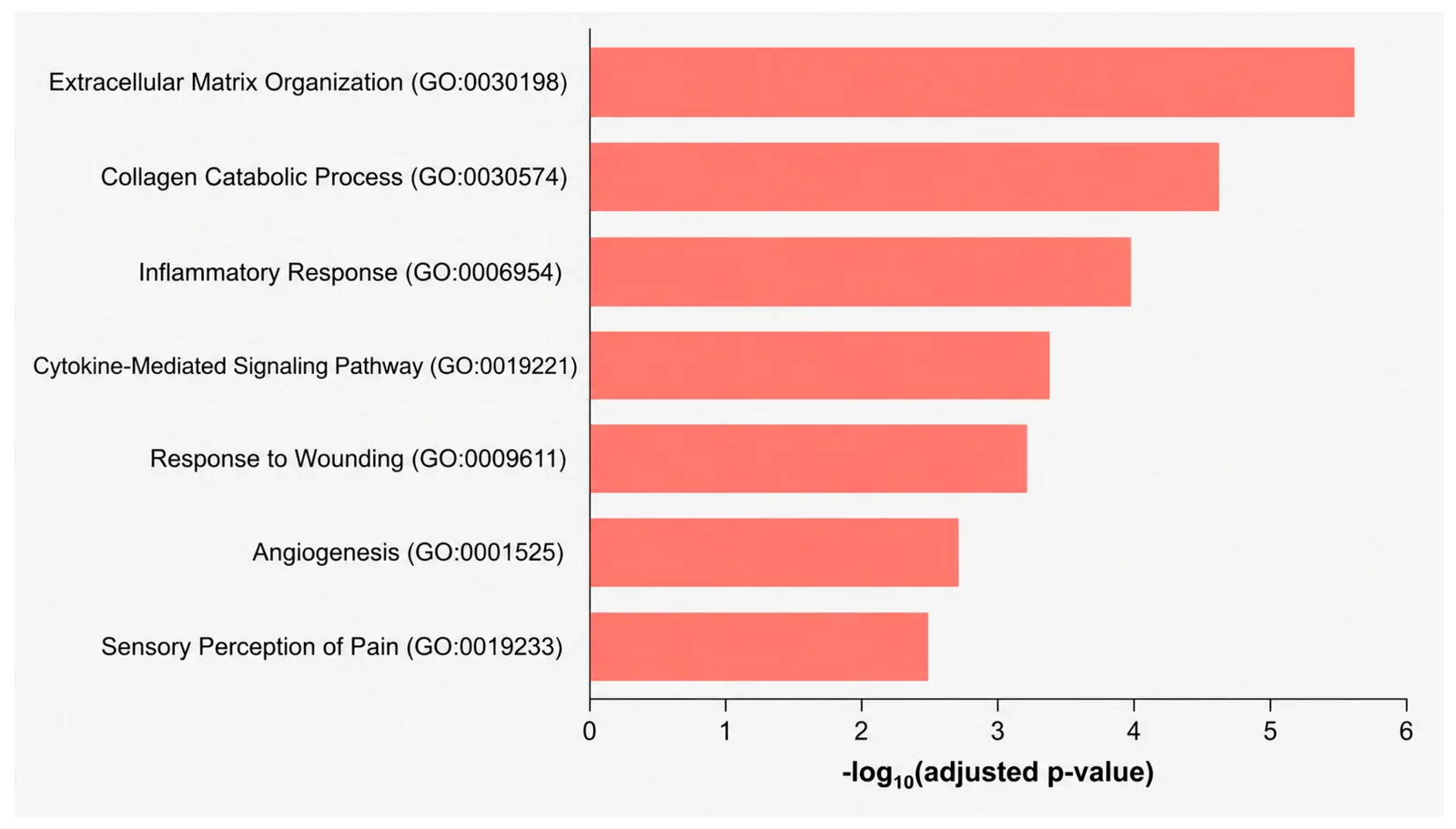
Functional annotation of the predefined 17-gene panel. The bar plot shows the significantly overrepresented Gene Ontology Biological Process terms reported in Supplementary Table S1a, displayed as −log10(Benjamini–Hochberg-adjusted *p*-values). Because the genes were selected a priori, the analysis was interpreted as functional annotation rather than as independent pathway discovery.

Functional annotation of the predefined 17-gene panel was performed using the Gene Ontology Biological Process, WikiPathways 2024 Human, KEGG, and BioPlanet databases. Because the panel was selected a priori to represent extracellular matrix remodeling, chronic inflammatory signaling, immune–stromal interactions, and sensory-associated processes, the enrichment analysis was interpreted exclusively as annotation of the panel’s biological composition rather than as discovery-level pathway identification. Gene Ontology Biological Process analysis identified significant overrepresentation of extracellular matrix organization (GO:0030198; FDR = 1.2 × 10^−6^), collagen catabolic process (GO:0030574; FDR = 3.4 × 10^−5^), inflammatory response (GO:0006954; FDR = 2.1 × 10^−4^), cytokine-mediated signaling pathway (GO:0019221; FDR = 4.8 × 10^−4^), response to wounding (GO:0009611; FDR = 7.9 × 10^−4^), angiogenesis (GO:0001525; FDR = 0.0021), and sensory perception of pain (GO:0019233; FDR = 0.0034) (Figure 2; Supplementary Table S1a). WikiPathways analysis identified OA chondrocyte hypertrophy (WP5373; FDR = 8.7 × 10^−5^), endochondral ossification (WP474; FDR = 1.3 × 10^−4^), endochondral ossification with skeletal dysplasias (WP4808; FDR = 2.9 × 10^−4^), IL-26 signaling (WP5347; FDR = 0.0018), and photodynamic therapy-induced NF-κB survival signaling (WP3617; FDR = 0.0031). KEGG analysis identified inflammatory mediator regulation of TRP channels (hsa04750; FDR = 6.1 × 10^−5^), Th17 cell differentiation (hsa04659; FDR = 2.7 × 10^−4^), cytokine–cytokine receptor interaction (hsa04060; FDR = 4.2 × 10^−4^), MAPK signaling pathway (hsa04010; FDR = 0.0019), and PI3K–Akt signaling pathway (hsa04151; FDR = 0.0038). BioPlanet analysis identified endochondral ossification (BP:000671; FDR = 1.1 × 10^−5^), extracellular matrix organization (BP:000112; FDR = 2.4 × 10^−5^), matrix metalloproteinase activity (BP:000298; FDR = 3.9 × 10^−5^), interleukin-mediated signaling (BP:000412; FDR = 0.0012), immune–stromal interactions (BP:000521; FDR = 0.0029), leptin-mediated modulation of immune responses (BP:000733; FDR = 0.0041), and oncostatin M signaling (BP:000812; FDR = 0.0053). These results indicate that the predefined panel represents biological domains related to extracellular matrix remodeling, inflammatory signaling, immune–stromal interactions, and sensory-associated processes. Database identifiers, overlapping genes, and adjusted *p*-values are provided in Supplementary Tables S1a–d.

### 3.3. Exploratory Network-Based Prioritization of Candidate miRNAs

**Figure 3:**
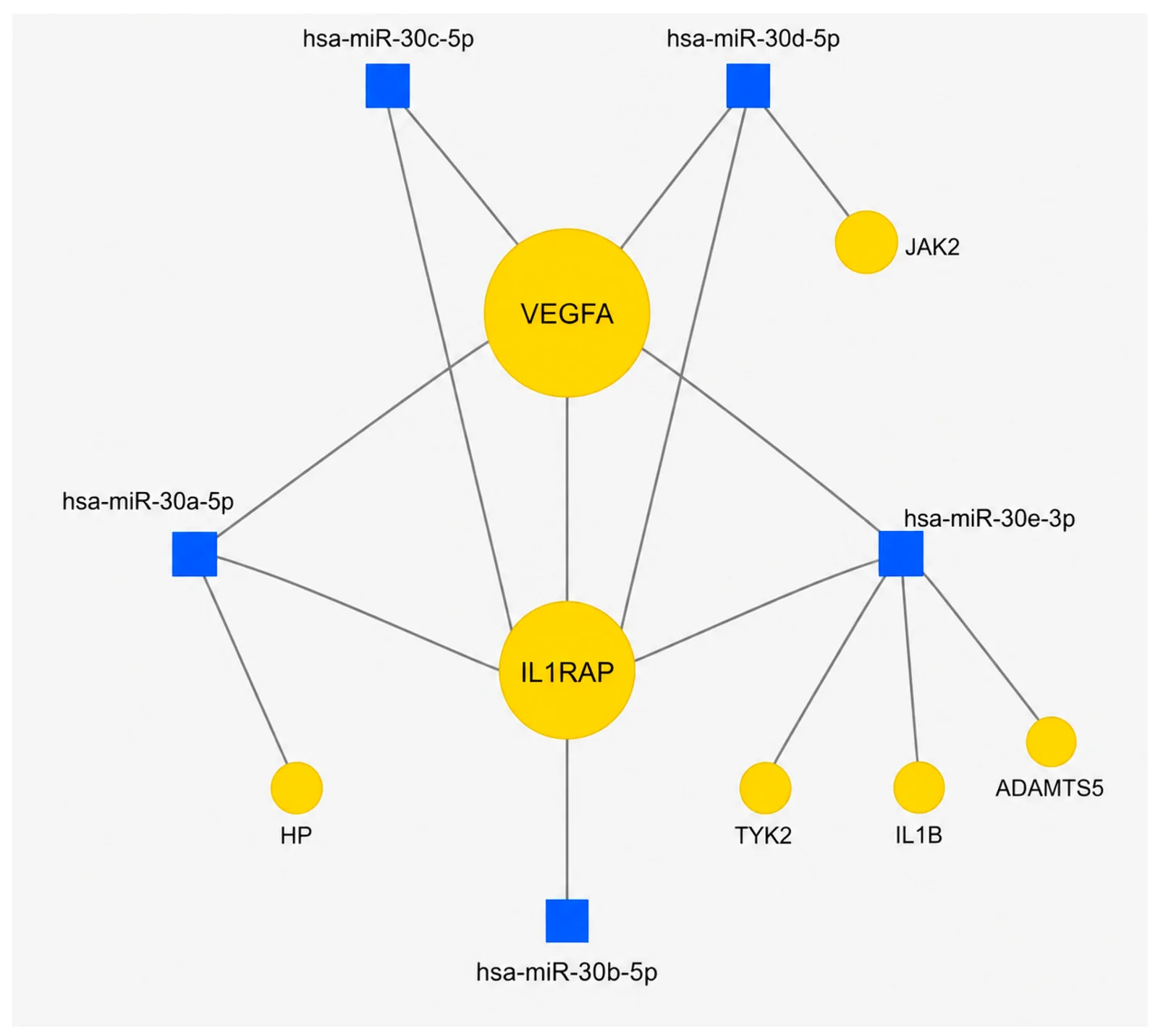
Focused database-derived miRNA-gene interaction network used for candidate prioritization. Blue square nodes represent the five experimentally measured mature miRNA strands, miR-30a-5p, miR-30b-5p, miR-30c-5p, miR-30d-5p, and miR-30e-3p. Yellow nodes represent genes from the predefined target panel that were connected to at least one of these miRNAs in the updated miRNet analysis. Edges represent interactions catalogued in miRTarBase as experimentally supported in previously reported experimental systems. The network was generated using human database annotations. These interactions may have been established in species or cellular contexts other than canine joint tissue and were not independently validated in the specimens analyzed in the present study.

An exploratory miRNA–gene interaction network was generated using miRNet and interactions catalogued in miRTarBase as experimentally supported in previously reported experimental systems. The updated analysis identified database-supported connections between the experimentally measured miR-30 mature strands and ADAMTS5, IL1B, IL1RAP, JAK2, TYK2, VEGFA, and HP. These genes represented the extracellular matrix remodeling, chronic inflammatory signaling, and immune–stromal interaction domains. The network was used solely to prioritize candidate miRNAs for targeted expression analysis. Because the underlying interactions may have been established in species, cell types, or experimental systems other than canine joint tissue, they were not interpreted as evidence of direct miRNA–mRNA regulation, functional coordination, compensatory activity, or a central regulatory role for the miR-30 family in the specimens analyzed. Following verification of assay identity, the final network interpretation was restricted to mature miRNA strands corresponding to those measured experimentally (Figure 3). Four of the five measured miR-30 mature strands showed a degree of at least two, whereas miR-30b-5p showed a single connection and was retained as part of the predefined miR-30 family panel. The complete initial candidate-miRNA ranking and connectivity metrics are provided in Supplementary Table S2a. Database-supported interactions between the experimentally measured miR-30 mature strands and genes of the predefined target panel, together with the corresponding experimental evidence, PMID, annotated tissue or experimental system, and database annotation species, are provided in Supplementary Table S2b.

### 3.4. RNA Quality Assessment and Technical Process Controls

Spectrophotometric analysis showed mean A260/A280 and A260/A230 absorbance ratios of 1.9 and 2.0, respectively, values consistent with limited protein and organic-compound carryover. RNA was recovered from all samples in amounts sufficient to provide the standardized input required for downstream RT-qPCR analyses. Qubit™ RNA IQ values ranged from 7 to 10, indicating relatively high RNA quality according to the Qubit™ RNA IQ assay. RNA IQ values were interpreted as assay-specific quality indicators and were not considered equivalent to electrophoretically determined RNA integrity numbers. Across samples, UniSp2 and UniSp4 amplified at Cq values of approximately 21 and 28, respectively, resulting in a difference of approximately 7 cycles. This difference was close to that expected from the 100-fold concentration difference between the two spike-ins and supported consistent extraction performance and recovery of the exogenous controls. It was not interpreted as evidence of endogenous miRNA integrity. UniSp6 amplified at a Cq value of approximately 19, indicating consistent reverse-transcription and amplification performance and no evidence of marked technical inhibition. The spike-in results were used exclusively as technical process-quality indicators and were not used for normalization of endogenous miRNA expression. No predefined exclusion threshold based on absorbance ratios or RNA IQ was applied, and no specimen was excluded because all samples provided sufficient RNA for the standardized RT-qPCR input.

### 3.5. Selection of Endogenous Reference miRNAs and Genes

The expression stability of three candidate reference miRNAs, miR-26a-5p, miR-103a-3p, and miR-186-5p, and three candidate reference genes, ACTB, GAPDH, and RPS18, was evaluated across the complete study sample set [n = 16] using four statistical approaches: the comparative ΔCq method [31], BestKeeper [32], NormFinder [33], and GeNorm [34]. The algorithm-specific results were integrated using RefFinder to obtain an overall stability ranking [35]. Although the individual algorithms produced partially different rankings, the integrated RefFinder analysis identified miR-103a-3p and ACTB as the highest-ranked candidates within the miRNA and mRNA reference panels, respectively. Accordingly, miR-103a-3p and ACTB were selected for normalization of miRNA and mRNA expression data, respectively (Table 4). The algorithm-specific rankings are reported in Table 4. These rankings apply specifically to the candidate reference miRNAs and genes evaluated in the present sample set and should not be interpreted as evidence of universal reference-gene stability across other tissues or experimental conditions.

### 3.6. miRNA Expression Profiling

Analysis of the miR-30 family revealed a consistent osteoarthritis-associated expression pattern across comparisons. Compared with control joint tissue (C-JT), OA samples showed significant downregulation of miR-30b-5p and upregulation of miR-30c-5p, miR-30d-5p, and miR-30e-3p, whereas miR-30a-5p did not differ significantly (Table 5).

**Figure 4:**
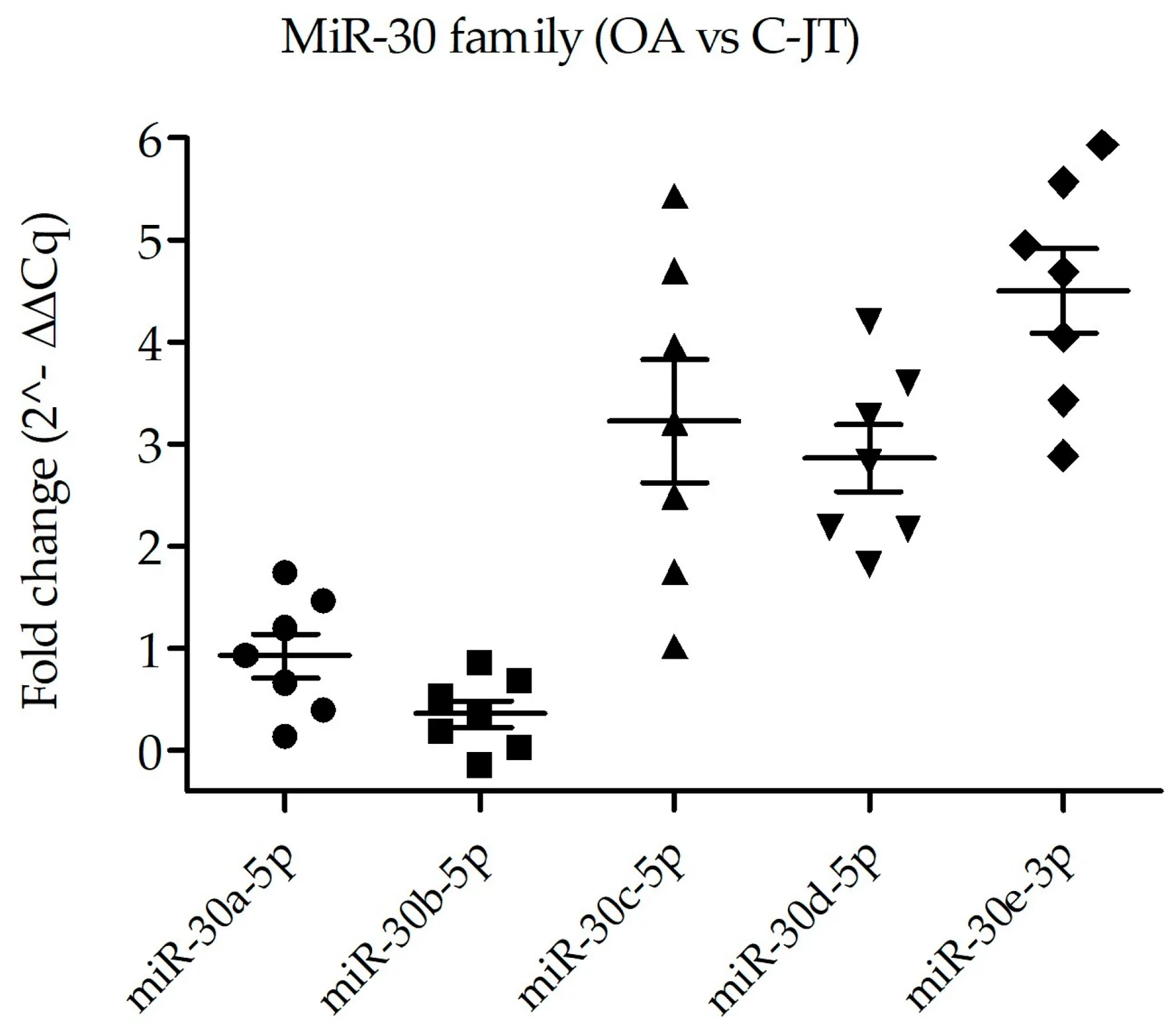
Relative expression of miR-30 family members in osteoarthritic (OA) samples compared with control joint tissue (C-JT) samples. Relative expression levels were calculated using the 2^−ΔΔCq method and are presented as fold changes relative to the C-JT group, which was used as the calibrator (fold change = 1). Individual data points represent biological replicates (n = 7), and horizontal bars indicate the mean ± SEM. Fold-change values < 1 indicate downregulation, whereas values > 1 indicate upregulation in OA samples relative to C-JT.

The 2^−ΔΔCq fold-change representation was consistent with the statistical analysis performed on ΔCq values, showing the same directional expression pattern relative to the C-JT calibrator (fold change = 1; Figure 4).

### 3.7. Gene Expression Profiling

Gene expression analysis showed differential expression patterns across genes involved in extracellular matrix remodeling, inflammatory signaling, immune–stromal interactions, and neuroinflammatory pathways in OA samples compared with C-JT. Among genes involved in extracellular matrix remodeling, ADAMTS5, MMP2, MMP13, and COL2A1 were significantly upregulated in OA samples, whereas MMP9 did not differ significantly. Among genes involved in immune–stromal interaction, SPARC, HP, VEGFA, and IGFBP6 were significantly upregulated. ASIC3 was also significantly upregulated, whereas NGFB and NTRK1 showed no significant differences. Conversely, none of the genes associated with chronic inflammatory signalling, including IL1B, IL1R1, IL1RAP, JAK2, and TYK2, differed significantly between OA and C-JT samples (Table 6).

**Figure 5:**
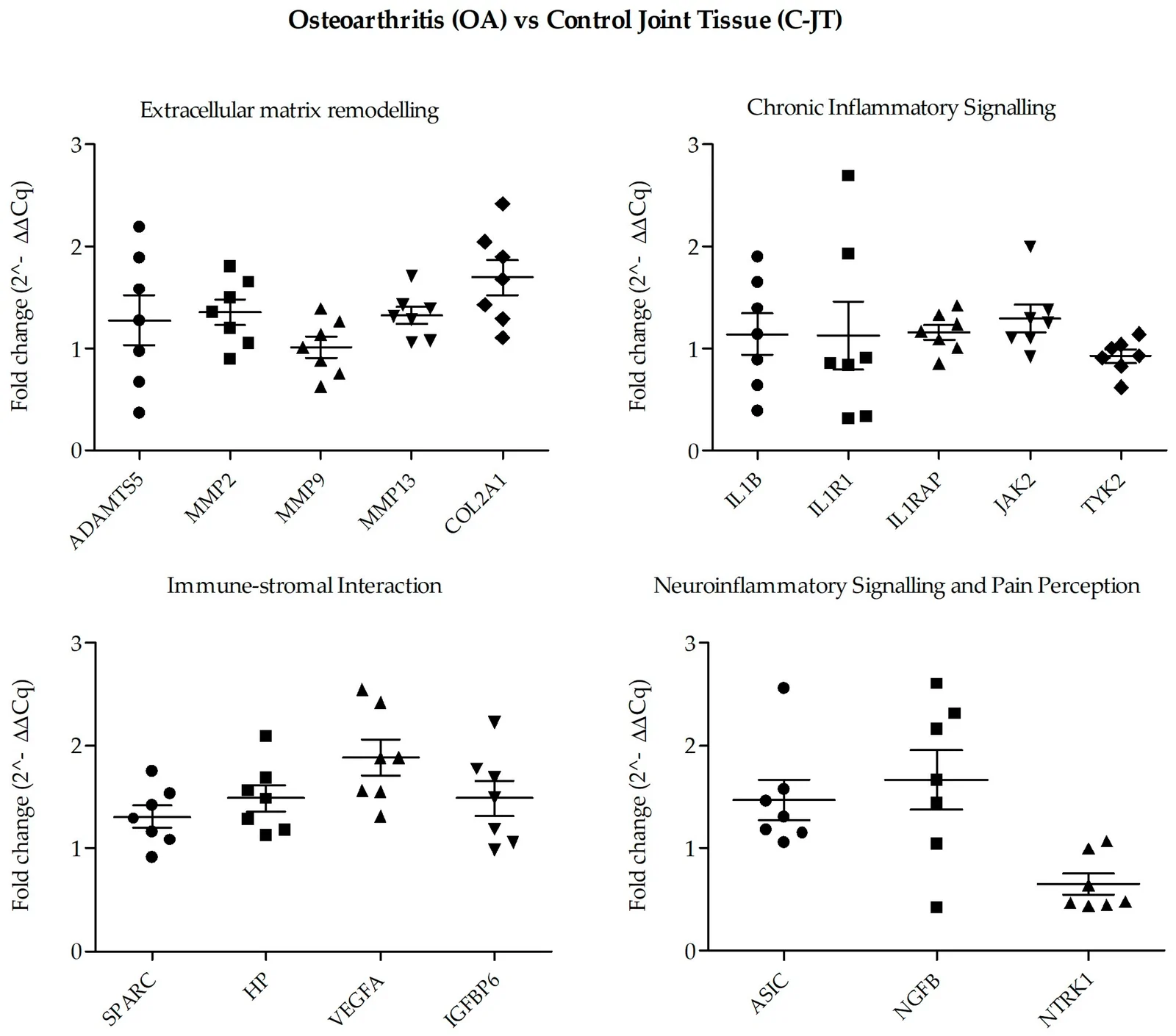
Relative expression of genes involved in extracellular matrix remodeling, chronic inflammatory signaling, immune–stromal interactions, and neuroinflammatory and sensory-associated processes in osteoarthritic joint tissues (OA; n = 7) compared with control joint tissues (C-JT; n = 5). All 17 genes were evaluated in all biological specimens from both groups. Relative expression levels were calculated using the 2^−ΔΔCq method and are presented as fold changes relative to the C-JT group, which was used as the calibrator (fold change = 1). Individual data points represent the seven OA biological specimens, and horizontal bars indicate the mean ± SEM. Fold-change values < 1 indicate lower expression, whereas values > 1 indicate higher expression in OA relative to C-JT.

Relative expression analysis using the 2^−ΔΔCq method was consistent with the statistical analysis performed on ΔCq values, showing the same directional expression pattern relative to the C-JT calibrator (fold change = 1; Figure 5).

## 4. Discussion

The present study compared anatomically comparable joint capsule specimens containing synovial membrane from dogs with established OA and control joint tissues without radiographic or histopathological evidence of OA. The primary OA versus C-JT analysis identified concurrent but distinct alterations in selected mRNAs and miR-30 family members. Because the study was cross-sectional and based on bulk FFPE tissue, these findings are interpreted as OA-associated expression patterns rather than as evidence of pathway activation, direct miRNA–mRNA regulation, compensatory activity, or disease progression.

Within the extracellular matrix domain, OA tissues showed significantly higher expression of ADAMTS5, MMP2, MMP13, and COL2A1, whereas MMP9 did not differ significantly between groups. The coexistence of changes in protease-related and structural transcripts is consistent with altered extracellular matrix turnover within the capsulosynovial compartment [9]. However, transcript abundance alone does not demonstrate proteolytic activity, matrix degradation, or an effective reparative response. In particular, the increase in COL2A1 should not be interpreted as evidence of cartilage repair because articular cartilage was not the tissue subjected to molecular analysis.

The expression profile of the inflammatory and immune–stromal domains was similarly heterogeneous. IL1B, IL1R1, IL1RAP, JAK2, and TYK2 did not differ significantly between OA and C-JT after false discovery rate correction. Therefore, the present data do not provide transcriptional evidence of broad activation of the selected inflammatory signaling pathways. In contrast, SPARC, HP, VEGFA, and IGFBP6 were significantly more highly expressed in OA tissues. These differences identify a tissue-response profile involving stromal, vascular, and extracellular microenvironment-related transcripts [10], but they do not establish the cellular sources of these molecules or demonstrate corresponding changes in protein abundance or biological activity.

Within the sensory-associated domain, ASIC3 expression was significantly higher in OA tissues, whereas NGFB and NTRK1 were unchanged. ASIC3 has been implicated in the responses of joint-innervating sensory neurons to acidic and inflammatory microenvironments [11,37]. Nevertheless, its increased transcript abundance in capsulosynovial tissue should be interpreted only as a sensory-associated molecular difference. It does not demonstrate nociceptor activation, establish the cellular origin of ASIC3 expression, or provide a direct measure of pain severity. Similarly, the absence of significant NGFB and NTRK1 differences does not exclude involvement of this signaling axis at the protein, cellular, or functional level [38].

The functional annotation analyses reflected extracellular matrix, inflammatory, immune–stromal, and sensory-associated domains. These findings were expected to some extent because the 17 genes had been selected a priori to represent these biological processes. Accordingly, the enrichment results describe the biological composition of the targeted panel and should not be interpreted as independent discovery of OA pathways or as evidence that the corresponding pathways were functionally activated in the analyzed tissues.

The miR-30 family exhibited a divergent expression pattern. miR-30b-5p was significantly downregulated, whereas miR-30c-5p, miR-30d-5p, and miR-30e-3p were significantly upregulated; miR-30a-5p did not differ between OA and C-JT. This divergence indicates that the measured miR-30 family members should not be interpreted as a single, coordinately regulated unit. The opposite direction observed for miR-30b-5p may reflect family-member-specific expression, processing, stability, cellular origin, or context-dependent activity [15,16]. However, the finding remains descriptive because of the small sample size and the absence of cell-resolved or functional validation.

The absence of a significant difference in miR-30a-5p expression is noteworthy because this miRNA showed high connectivity in the database-derived interaction network and has previously been implicated in ADAMTS5 regulation in human OA. However, network connectivity reflects previously reported interactions and was used here only for candidate prioritization; it does not predict differential expression in canine capsulosynovial tissue. The lack of a significant miR-30a-5p difference in the present cohort may reflect species-specific regulation, tissue-compartment and cellular-composition differences, context-dependent miRNA expression, or the limited statistical power of the small cohort. Thus, network connectivity and differential expression should be regarded as distinct analytical levels, and the database-based ranking should not be interpreted as a predictor of in vivo expression change.

Although miRNAs are commonly associated with reduced target expression, an inverse relationship between miRNA and mRNA abundance cannot be assumed from concurrent bulk-tissue measurements. In mammalian experimental systems, canonical miRNA-mediated repression frequently involves reduced target mRNA abundance [39]. Nevertheless, the absence of an inverse expression pattern does not exclude regulation, just as concurrent increases do not demonstrate direct positive regulation. The joint capsule and synovial membrane contain heterogeneous stromal, vascular, immune, and other cell populations [40]; consequently, the measured miRNAs and mRNAs may not have originated from the same cells. In addition, the miRNA–gene interactions used for candidate prioritization were documented in previously reported species, cell types, or experimental systems and were not demonstrated directly in the canine tissues analyzed here.

Therefore, the concurrent increases in miR-30c-5p, miR-30d-5p, miR-30e-3p, and several OA-associated transcripts do not demonstrate canonical negative regulation, miRNA-mediated compensation, direct positive regulation, impaired miRNA processing, saturation of the miRNA pathway, or translational activation. Noncanonical miRNA-mediated translational activation has been demonstrated in specialized experimental systems, including nonadenylated target mRNAs in a mammalian cell-free system [41]. However, the present study provides no evidence that this mechanism operates in canine osteoarthritic joint tissue. Similarly, saturation of the cellular miRNA machinery has been demonstrated under experimentally induced high small-RNA expression [42], but the abundance or activity of DROSHA, DICER, AGO proteins, and other components of the miRNA-processing and effector machinery was not evaluated here. Altered processing or pathway saturation can therefore be mentioned only as speculative alternatives that require direct investigation.

The network analysis should consequently be regarded as a hypothesis-generating strategy for candidate prioritization. It identifies previously reported connections between the exact mature miRNA strands measured experimentally and selected transcripts, but it does not establish that these interactions occur in canine capsulosynovial tissue. Within-sample miRNA–mRNA correlation analysis was not pursued because the small anatomically matched cohort and the number of candidate interactions would not support reliable mechanistic inference. Even a statistically significant correlation would not, in the absence of perturbation or reporter experiments, demonstrate direct regulation.

Several limitations should be considered. The final primary cohort was small, comprising seven OA and five C-JT specimens, which limits statistical precision and the ability to detect modest differences despite the use of Welch’s tests, confidence intervals, and false discovery rate correction. Accordingly, significant findings may have imprecise effect-size estimates and require confirmation in larger cohorts, whereas non-significant findings should not be interpreted as evidence of absence of a biological difference because modest effects may not have been detectable with the present sample size. The groups were not matched for age, sex, or sampling site, and these variables should therefore be considered potential residual confounders. In addition, the retrospective design limited the availability and uniformity of information regarding specimen acquisition, fixation duration, post-mortem interval, detailed separation of OA grades, and standardized synovitis scoring. Although surgical and necropsy specimens were processed within the routine FFPE workflow of the same diagnostic laboratory, systematic differences in preanalytical handling between OA and C-JT cannot be excluded and may have contributed to the observed expression differences, particularly for RNA measurements in FFPE tissue. The use of FFPE material may have reduced sensitivity for subtle expression differences. Moreover, bulk-tissue analysis did not resolve the contribution of individual cell populations. Protein measurements, spatial or single-cell analyses, target-reporter assays, and miRNA perturbation experiments were not performed. Complete assay-specific amplification efficiencies, standard curves, and amplicon sequencing or gel-based confirmation were unavailable for some assays originally developed for non-canine species. Although absolute quantification was not performed in the present study, target-specific differences in amplification efficiency or specificity may have affected the accuracy and magnitude of the relative ΔCq-based expression estimates, particularly for non-canine assays such as those used for ADAMTS5 and VEGFA. Findings obtained with the non-canine assays should therefore be interpreted cautiously and require confirmation using assays experimentally validated in canine tissue. These limitations preclude causal, cellular, longitudinal, and therapeutic interpretations.

Future studies should validate these expression patterns in larger, prospectively characterized and anatomically standardized canine cohorts. Cell-resolved and spatial approaches will be required to determine whether the altered miRNAs and transcripts are expressed within the same cellular compartments. Protein-level measurements, reporter assays, and gain- or loss-of-function experiments will be necessary to test the database-supported miRNA–gene interactions directly. Within these limits, the present findings identify concurrent OA-associated alterations in selected matrix-remodeling, immune–stromal, and sensory-associated transcripts together with miR-30 family members and provide a focused set of candidates for subsequent mechanistic investigation.

## 5. Conclusions

Canine osteoarthritic capsulosynovial tissues showed concurrent alterations in selected mRNAs and miR-30 family members compared with anatomically comparable control joint tissues. The expression profile involved matrix-remodeling, immune–stromal, and sensory-associated transcripts, while miR-30b-5p changed in the opposite direction to miR-30c-5p, miR-30d-5p, and miR-30e-3p. These findings identify OA-associated molecular patterns but do not demonstrate pathway activation, direct miRNA–mRNA regulation, compensatory mechanisms, or disease progression. Larger, cell-resolved and functional studies are required to clarify the biological significance of these candidate interactions.

## Figures and Tables

**Table 1:** Study population and specimen characteristics. (**a**) Demographic and anatomical characteristics of the 12 biologically independent canine tissue specimens included in the study. (**b**) Radiographic grade and histopathological classification of the osteoarthritic joint tissues and control joint tissues. Abbreviations: M, male; F, female; SM, synovial membrane.

(**a**)						
**Tissue category**	**Breed**	**Age (years)**	**Sex**	**Sampling site**	**Sample ID**	**Animal ID**
**Osteoarthritic joint tissue (OA)**	Mixed breed	11	M	Knee joint capsule with SM	OA-1	Dog-1
	Mixed breed	11	M	Knee joint capsule with SM	OA-2	Dog-2
	Mixed breed	10	M	Knee joint capsule with SM	OA-3	Dog-3
	German Shepherd	8	M	Elbow joint capsule with SM	OA-4	Dog-4
	German Shepherd	7	F	Knee joint capsule with SM	OA-5	Dog-5
	Mixed breed	15	M	Knee joint capsule with SM	OA-6	Dog-6
	Golden Retriever	8	M	Elbow joint capsule with SM	OA-7	Dog-7
**Control joint tissue (C-JT)**	Mixed breed	5	M	Knee joint capsule with SM	C-JT1	Dog-8
	Mixed breed	15	M	Knee joint capsule with SM	C-JT2	Dog-9
	Mixed breed	9	F	Elbow joint capsule with SM	C-JT3	Dog-10
	Mixed breed	6	F	Knee joint capsule with SM	C-JT4	Dog-11
	Mixed breed	5	M	Knee joint capsule with SM	C-JT5	Dog-12
(**b**)						
**Sample ID**	**Animal ID**	**Radiographic grade**		**Histopathological diagnosis**		
OA-1	Dog-1	Grade 2–3		Histopathologically consistent with OA		
OA-2	Dog-2	Grade 2–3		Histopathologically consistent with OA		
OA-3	Dog-3	Grade 2–3		Histopathologically consistent with OA		
OA-4	Dog-4	Grade 2–3		Histopathologically consistent with OA		
OA-5	Dog-5	Grade 2–3		Histopathologically consistent with OA		
OA-6	Dog-6	Grade 2–3		Histopathologically consistent with OA		
OA-7	Dog-7	Grade 2–3		Histopathologically consistent with OA		
C-JT1	Dog-8	Grade 0		No histopathological evidence of OA		
C-JT2	Dog-9	Grade 0		No histopathological evidence of OA		
C-JT3	Dog-10	Grade 0		No histopathological evidence of OA		
C-JT4	Dog-11	Grade 0		No histopathological evidence of OA		
C-JT5	Dog-12	Grade 0		No histopathological evidence of OA		

**Table 2:** TaqMan Gene Expression Assays used for RT-qPCR analysis. The table reports the gene symbol, TaqMan Gene Expression Assay ID, reference sequence accession number, exon location, and amplicon length in base pairs. Species prefixes in the assay IDs indicate “Hs” for Homo sapiens, “Oa” for Ovis aries, “Oc” for Oryctolagus cuniculus, and “Cf” for Canis familiaris. For assays originally designed for non-canine species, sequence compatibility was assessed by comparison of the annotated target/exon region with the corresponding canine transcript reported in Table 2, and homology of the target region was additionally confirmed through technical consultation. Because complete assay-specific efficiency and amplicon-validation data were not available, the use of these assays is acknowledged as a methodological limitation. The table includes 17 target genes and three candidate endogenous reference genes. Target genes are shown in white and candidate reference genes in gray. The dash indicates that exon-location information was not available for the ASIC3 assay.

Gene Symbol	TaqMan ID	Sequence ID	Exon	Amplicon (bp)
ADAMTS5	Hs01095518_m1	NM_007038.4	1–2	68
MMP2	Cf01548727_m1	XM_014109407.1	1–2	65
MMP9	Cf02621845_m1	NM_001003219.2	12–13	59
MMP13	Cf02741638_m1	XM_005619857.3	7–8	99
COL2A1	Cf02622868_m1	NM_001006951.1	7–8	102
IL1B	Cf02671952_m1	NM_001037971.1	4–5	70
IL1R1	Cf02647245_m1	XM_005625999.3	5–6	149
IL1RAP	Oc06776406_m1	XM_008266655.2	2–3	65
JAK2	Cf02685928_m1	XM_014117832.2	6–7	177
TYK2	Cf02633362_m1	XM_022407058.1	24–25	74
SPARC	Cf02661382_m1	XM_005619272.3	5–6	67
VEGFA	Oa04653812_m1	NM_001025110.1	2–3	118
HP	Cf02630391_m1	XM_845903.5	3–4	71
IGFBP6	Cf02664455_g1	XM_853380.5	3–4	105
NGFB	Cf02697134_s1	NM_001194950.1	1–1	161
NTRK1	Cf02676040_m1	XM_022421240.1	3–4	114
ASIC3	Cf02703543_m1	XM_022403869.1	-	121
ACTB	Cf04931159_m1	NM_001195845.2	1–2	53
GAPDH	Cf04419463	NM_001003142.2	5–6	54
RPS18	Cf02681523_g1	NM_001048082.1	3–4	160

**Table 3:** miRCURY LNA PCR primer assays used for miRNA expression analysis. The table reports the assay designation, miRBase accession number, mature miRNA sequence, and QIAGEN catalog number. Assay designations include mature miRNAs annotated in Canis familiaris (cfa), Homo sapiens (hsa), and Gallus gallus (gga). Species-specific assay names were retained as provided by the manufacturer and cross-referenced with the corresponding mature miRNA sequences. The table includes five target miRNAs (cfa-miR-30a-5p, hsa-miR-30b-5p, gga-miR-30c-5p, cfa-miR-30d-5p, and hsa-miR-30e-3p), three candidate endogenous reference miRNAs (hsa-miR-26a-5p, hsa-miR-103a-3p, and hsa-miR-186-5p), and three exogenous spike-in controls (UniSp2, UniSp4, and UniSp6). UniSp2 and UniSp4 were used to monitor RNA-extraction performance, whereas UniSp6 was used to monitor reverse-transcription and amplification performance. The spike-in controls were not used for normalization of endogenous miRNA expression. A dash indicates that the miRBase accession number or mature miRNA sequence is not applicable to the synthetic spike-in controls.

miRNA	miRBase Accession	Mature miRNA Sequence	QIAGEN Catalog Number
cfa-miR-30a-5p	MIMAT0006604	UGUAAACAUCCUCGACUGGAAGC	YP02101182
hsa-miR-30b-5p	MIMAT0000420	UGUAAACAUCCUACACUCAGCU	YP00204765
gga-miR-30c-5p	MIMAT0001137	UGUAAACAUCCUACACUCUCAGCU	YP00205948
cfa-miR-30d-5p	MIMAT0006616	UGUAAACAUCCCCGACUGGAAGCU	YP02118689
hsa-miR-30e-3p	MIMAT0000693	CUUUCAGUCGGAUGUUUACAGC	YP00204410
hsa-miR-26a-5p [29]	MIMAT0000082	UUCAAGUAAUCCAGGAUAGGCU	YP00206023
hsa-miR-103a-3p [30]	MIMAT0000101	AGCAGCAUUGUACAGGGCUAUGA	YP00204063
hsa-miR-186-5p [29]	MIMAT0000456	CAAAGAAUUCUCCUUUUGGGCU	YP00206053
UniSp2	-	-	YP00203950
UniSp4	-	-	YP00203953
UniSp6	-	-	YP00203954

**Table 4:** Stability ranking of candidate endogenous reference miRNAs and genes. The table reports the ranking generated by the comparative ΔCq method, BestKeeper, NormFinder, and geNorm, together with the integrated RefFinder ranking. miR-103a-3p and ACTB ranked first among the three candidate reference miRNAs and three candidate reference genes, respectively, and were therefore selected for normalization of the corresponding RT-qPCR data. Rank 1 indicates the highest expression stability within each candidate set.

	EC Gene			EC miRNA		
	Rank 1	Rank 2	Rank 3	Rank 1	Rank 2	Rank 3
Delta Ct	ACTB	GAPDH	RPS18	miR-103a-3p	miR-26a-5p	miR-186-5p
BestKeeper	GAPDH	ACTB	RPS18	miR-103a-3p	miR-26a-5p	miR-186-5p
NormFinder	ACTB	GAPDH	RPS18	miR-103a-3p	miR-26a-5p	miR-186-5p
GeNorm	ACTB	GAPDH	RPS18	miR-26a-5p/ miR-103a-3p	miR-186-5p	
RefFinder	ACTB	GAPDH	RPS18	miR-103a-3p	miR-26a-5p	miR-186-5p

**Table 5:** Differential expression of miR-30 family members between osteoarthritic (OA) and control joint tissue (C-JT) samples. All five miRNAs were evaluated in seven OA and five C-JT biological specimens. Statistical comparisons were performed on ΔCq values using two-sided unpaired Welch’s *t*-tests. The estimated effect is reported as the difference between the mean ΔCq values of the OA and C-JT groups (OA − C-JT) ± standard error (SE), together with its 95% confidence interval. Because lower ΔCq values indicate higher normalized expression, a negative mean difference corresponds to higher expression in OA, whereas a positive mean difference corresponds to lower expression in OA. Raw *p*-values were adjusted using the Benjamini–Hochberg procedure to control the false discovery rate. Statistical significance was assessed using FDR-adjusted *p*-values: ns, FDR ≥ 0.05; * FDR < 0.05; ** FDR < 0.01. ↑ OA and ↓ OA indicate significantly higher and lower expression, respectively, in OA samples relative to C-JT; — indicates no significant difference.

Osteoarthritis (OA) vs. Control Joint Tissue (C-JT)						
miRNA	Raw *p*-Value	Mean ΔCq Difference ± SE	95% CI	FDR	Significance	Direction of Change
miR-30a-5p	0.3846	0.580 ± 0.631	−0.875 to 2.035	0.3846	ns	—
miR-30b-5p	0.0039	2.200 ± 0.572	0.906 to 3.495	0.0098	**	↓ OA
miR-30c-5p	0.0098	−1.421 ± 0.436	−2.406 to −0.436	0.0123	*	↑ OA
miR-30d-5p	0.0018	−1.458 ± 0.300	−2.168 to −0.747	0.0090	**	↑ OA
miR-30e-3p	0.0073	−1.858 ± 0.540	−3.078 to −0.638	0.0122	*	↑ OA

**Table 6:** Differential expression of selected genes between osteoarthritic (OA) and control joint tissue (C-JT) samples. Genes are grouped according to their predefined functional category. All seventeen genes were evaluated in seven OA and five C-JT biological specimens. Statistical comparisons were performed on ΔCq values using two-sided unpaired Welch’s *t*-tests. The estimated effect is reported as the difference between the mean ΔCq values of the OA and C-JT groups (OA − C-JT) ± standard error (SE), together with its 95% confidence interval. Because lower ΔCq values indicate higher normalized expression, a negative mean difference corresponds to higher expression in OA, whereas a positive mean difference corresponds to lower expression in OA. Raw *p*-values were adjusted using the Benjamini–Hochberg procedure to control the false discovery rate. Statistical significance was determined using FDR-adjusted *p*-values: ns, FDR ≥ 0.05; * FDR < 0.05. ↑ OA indicate significantly higher expression, in OA samples relative to C-JT; — indicates no significant difference.

Osteoarthritis (OA) vs. Control Joint Tissue (C-JT)							
Functional Category	Gene	Mean ΔCq Difference ± SE	95% CI	Raw *p*-Value	FDR	Significance	Direction of Change
Extracellular matrix remodelling	ADAMTS5	−0.2454 ± 0.07698	−0.4195 to −0.07128	0.0110	0.0312	*	↑ OA
	MMP2	−0.4242 ± 0.1491	−0.7614 to −0.08696	0.0192	0.0326	*	↑ OA
	MMP9	0.2709 ± 0.2161	−0.2180 to 0.7598	0.2416	0.2934	ns	—
	MMP13	−0.3821 ± 0.1117	−0.6397 to −0.1245	0.0091	0.0310	*	↑ OA
	COL2A1	−0.7071 ± 0.1850	−1.160 to −0.2544	0.0087	0.0310	*	↑ OA
Chronic inflammatory signalling	IL1B	−0.1209 ± 0.09234	−0.3393 to 0.09749	0.2318	0.2934	ns	—
	IL1R1	0.09490 ± 0.4195	−0.8972 to 1.087	0.8275	0.8792	ns	—
	IL1RAP	−0.2062 ± 0.1181	−0.4734 to 0.06100	0.1148	0.1626	ns	—
	JAK2	−0.3267 ± 0.2198	−0.8240 to 0.1706	0.1714	0.2285	ns	—
	TYK2	0.1398 ± 0.1877	−0.2847 to 0.5643	0.4753	0.5387	ns	—
Immune–stromal interaction	SPARC	−0.1753 ± 0.04162	−0.2771 to −0.07346	0.0056	0.0310	*	↑ OA
	HP	−0.5369 ± 0.1583	−0.9019 to −0.1719	0.0095	0.0310	*	↑ OA
	VEGFA	−0.8684 ± 0.1848	−1.286 to −0.4504	0.0011	0.0187	*	↑ OA
	IGFBP6	−0.5059 ± 0.1682	−0.8864 to −0.1254	0.0148	0.0315	*	↑ OA
Neuroinflammatory signalling and pain perception	ASIC3	−0.4846 ± 0.09523	−0.7176 to −0.2516	0.0022	0.0187	*	↑ OA
	NGFB	−0.5093 ± 0.3822	−1.374 to 0.3552	0.2154	0.2934	ns	—
	NTRK1	0.7408 ± 0.4241	−0.2623 to 1.744	0.1242	0.1656	ns	—

## Data Availability

The original contributions presented in this study are included in the article/Supplementary Materials. Further inquiries can be directed to the corresponding author.

## Supplementary Materials

The following supporting information can be downloaded at https://www.mdpi.com/article/10.3390/biomedicines14092024/s1, Figure S1: Representative radiographic features of normal and osteoarthritic canine joints; Figure S2a: Functional annotation of the predefined gene panel using WikiPathways; Figure S2b: Functional annotation of the predefined gene panel using KEGG; Figure S2c: Functional annotation of the predefined gene panel using BioPlanet; Table S1a: Gene Ontology Biological Process annotation of the predefined gene panel; Table S1b: WikiPathways annotation of the predefined gene panel; Table S1c: KEGG annotation of the predefined gene panel; Table S1d: BioPlanet annotation of the predefined gene panel; Table S2a: Initial ranking and connectivity metrics of candidate miRNAs identified through miRNet; Table S2b: Database-supported interactions between the experimentally measured miR-30 mature strands and genes included in the predefined target panel.
