# Supplementary material for: Concurrent Alterations in Selected mRNAs and miR-30 Family Members in Canine Osteoarthritic Joint Tissues
Source: Biomedicines. 2026 Sep 9;14(9):2024. doi: 10.3390/biomedicines14092024 (PMC13604073; doi:10.3390/biomedicines14092024)
Supplement: Supplementary file 1 [file biomedicines-14-02024-s001.zip › Supplemental material S1.pdf]

Supplemental Material S1

Classification and Eligibility Criteria for OA andC-JT This retrospective study included archived tissue samples obtained during surgical procedures or post-mortem examinations. Available clinical, radiographic, and histopathological records were reviewed for each case to assess study eligibility. Because of the retrospective study design, the nature and completeness of the available information varied among cases.

Specimens were classified into two predefined categories:

1. OA: joint tissues from joints with established osteoarthritis;
2. C-JT: control joint tissues from joints without evidence of osteoarthritis

Radiographic Diagnosis

When radiographs were available, they had been obtained either during the clinical diagnostic evaluation or in association with the post-mortem examination. Radiographic examinations included, where available, orthogonal projections appropriate to the joint examined, such as mediolateral and craniocaudal or ventrodorsal views.

Radiographic images were reviewed at the University of Perugia Veterinary Teaching Hospital using a commercial Digital Imaging and Communications in Medicine viewer (OsiriX MD v13.0, Pixmeo SARL, Switzerland).

Radiographic records for OA and C-JT joints were graded according to criteria adapted from the Kellgren–Lawrence scoring system [1] and veterinary radiographic scoring schemes. The key radiographic features of OA evaluated included osteophyte formation, subchondral bone changes (including sclerosis), joint space narrowing, periarticular mineralization, joint effusion, and joint remodeling. Higher scores indicated more advanced osteoarthritic changes.

Radiographic grading was performed by a trained operator according to predefined criteria to ensure consistency in classification.

The radiographic grading criteria were defined as follows:

– Grade 0 (No OA): no radiographic evidence of OA; absence of osteophytes, normal subchondral bone, and preserved joint contours.

– Grade 1 (Mild OA): minimal osteoarthritic changes, characterized by small periarticular osteophytes without significant subchondral sclerosis or joint remodeling.

– Grade 2 (Moderate OA): clearly visible osteophyte formation accompanied by mild to moderate subchondral sclerosis and early joint remodeling.

– Grade 3 (Severe OA): marked osteophyte formation, pronounced subchondral sclerosis, joint margin irregularities, and advanced joint remodeling consistent with chronic OA.

Grade 0 joints were eligible for inclusion in the C-JT category when histopathological evaluation showed no evidence of OA or another relevant joint disorder. Joints classified as Grade 1 were excluded from the tissue analysis to reduce uncertainty associated with mild or early degenerative changes. Joints classified radiographically as Grade 2 or Grade 3 were eligible for inclusion in the OA category when the available clinicopathological findings were consistent with osteoarthritic disease.

Representative radiographs illustrating the radiographic appearance of C-JT and OA joints are shown in Figure SM1. Images highlight the structural changes used for radiographic classification and tissue selection.

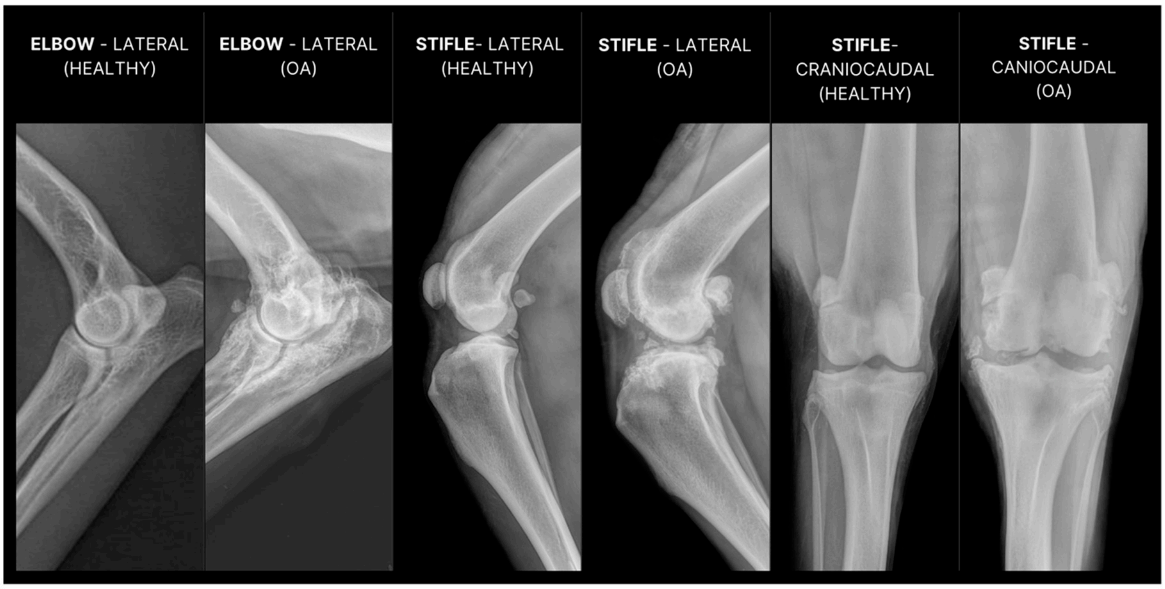

Figure S1. Representative radiographic features of normal and osteoarthritic canine joints. Representative radiographs of the elbow and stifle joints showing normal (Grade 0; C-JT group) and severe osteoarthritic changes (Grade 3, OA group). Osteoarthritic joints were characterized by marked osteophyte formation, subchondral sclerosis, joint space narrowing, periarticular mineralization, and advanced joint remodeling, whereas normal joints showed preserved joint contours and the absence of radiographic abnormalities. These representative images illustrate the radiographic criteria used for osteoarthritis grading and tissue selection.

Histopathological Diagnosis

Histopathological evaluation was performed by a board-certified veterinary pathologist (Prof. Leonardo Leonardi) using standardized morphological criteria. Joint tissues were eligible for inclusion in the OA category when their histopathological findings, interpreted together with the available clinical and radiographic information, were consistent with chronic osteoarthritic disease. This criterion also permitted the inclusion of OA joint tissues for which complete radiographic documentation was unavailable.

The principal histopathological features considered supportive of OA classification included:

- chronic hyperplastic lymphoplasmacytic and histiocytic synovitis with associated articular cartilage degeneration and mild osseous remodeling;
- synovial hyperplasia associated with moderate lymphoplasmacytic synovitis;
- chronic hyperplastic lymphoplasmacytic and macrophagic synovitis.

Joint tissues included in the C-JT category were required to show no histopathological evidence of OA, relevant degenerative changes, or another joint disease.

Exclusion Criteria

Based on the available clinical, radiographic, and histopathological records, cases or specimens were excluded when one or more of the following conditions were identified:

– Insufficient tissue preservation or, for post-mortem material, advanced decomposition

– Systemic infectious diseases that could interfere with miRNA or histopathological analyses

– Cases with a known history of intra-articular injections (when this information was available), due to their potential influence on synovial cellular composition and miRNA expression

– Joints with evidence of articular fractures or prior surgical procedures (e.g., surgically treated CCL rupture, arthrodesis, or total joint replacement)

– Neoplastic conditions involving joints or the musculoskeletal system

– Congenital joint abnormalities unrelated to OA

– Septic (infectious) arthritis

– Leishmaniosis or other systemic diseases known to affect joint integrity or inflammatory status

– Histopathological findings inconsistent with OA, including predominant neutrophilic synovitis, panniculitis, or lesions suggestive of immune-mediated, infectious, or systemic inflammatory arthropathies.

Joint tissues were excluded from the C-JT category if any available radiographic or histopathological finding indicated OA, degenerative joint disease, or another relevant articular disorder.

References

1. Kellgren, J.H.; Lawrence, J.S. Radiological Assessment of Osteo-Arthrosis. Annals of the Rheumatic Diseases 1957, 16, 494–502, doi:10.1136/ard.16.4.494.
