# Supplementary material for: Concurrent Alterations in Selected mRNAs and miR-30 Family Members in Canine Osteoarthritic Joint Tissues
Source: Biomedicines. 2026 Sep 9;14(9):2024. doi: 10.3390/biomedicines14092024 (PMC13604073; doi:10.3390/biomedicines14092024)
Supplement: Supplementary file 1 [file biomedicines-14-02024-s001.zip › Supplemental material S2.pdf]

Supplementary Material S2

Figure S2

To provide a functional annotation of the predefined gene panel investigated in this study, over-representation analysis was performed using pathway-based databases. The analysis identified pathway terms that were significantly overrepresented among the genes included in the panel. These results describe the functional categories represented by this predefined panel and should not be interpreted as establishing causal or disease-driving processes in OA. Pathways are reported according to their statistical ranking in the respective databases.

- WikiPathways enrichment [1] analysis using the WikiPathways 2024 Human database identified several significantly overrepresented pathways. The top statistically significant terms ranked by adjusted p-value included OA chondrocyte hypertrophy (WP5373), endochondral ossification (WP474), and endochondral ossification with skeletal dysplasias (WP4808). Additional enriched pathways included IL-26 signaling (WP5347) and photodynamic therapy-induced NF-κB survival signaling (WP3617). The top statistically significant pathways ranked by adjusted p-value are presented in Figure SM2a.

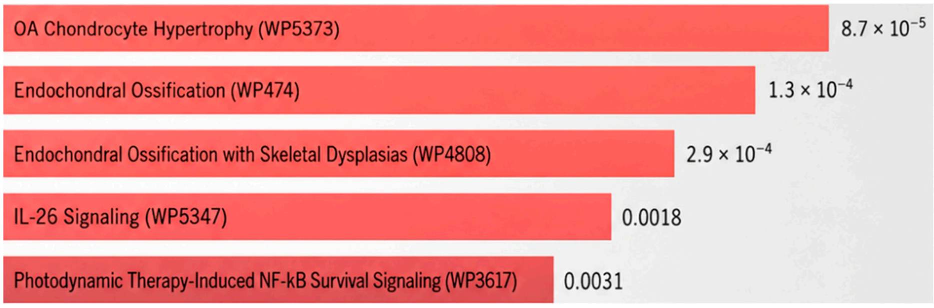

Figure S2a. Functional annotation of the predefined gene panel using WikiPathways. Bar plot showing the significantly overrepresented WikiPathways terms reported in Supplementary Table SM2b, ranked by Benjamini–Hochberg-adjusted p-value.

- KEGG pathway [2,3] enrichment analysis identified significant overrepresentation of pathways related to inflammatory mediator regulation of TRP channels, Th17 cell differentiation, cytokine–cytokine receptor interaction, and MAPK and PI3K–AKT signaling. The top statistically significant pathways ranked by adjusted p-value are presented in Figure SM2b.

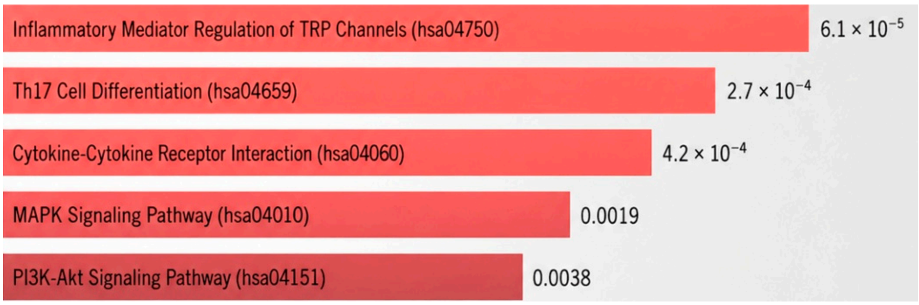

Figure S2b. Functional annotation of the predefined gene panel using KEGG. Bar plot showing the significantly overrepresented KEGG pathways reported in Supplementary Table SM2c, ranked by Benjamini–Hochberg-adjusted p-value.

BioPlanet enrichment analysis revealed significant overrepresentation of pathways associated with endochondral ossification, extracellular matrix organization, and matrix metalloproteinase activity, as well as interleukin-mediated

and immune system signaling. Additional enriched pathways included leptin-mediated modulation of immune responses and oncostatin M signaling. The top statistically significant pathways ranked by adjusted p-value are presented in Figure SM2c.

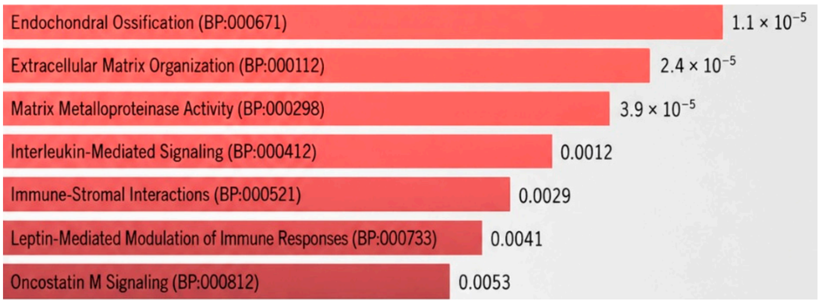

Figure S2c. Functional annotation of the predefined gene panel using BioPlanet. Bar plot showing the significantly overrepresented BioPlanet pathways reported in Supplementary Table SM2d, ranked by Benjamini–Hochberg-adjusted p-value.

Table S1

The statistically significant terms identified through Gene Ontology Biological Process, WikiPathways 2024 Human, KEGG, and BioPlanet analyses are reported in Supplementary Tables SM2a–d. Terms are presented according to their adjusted p-values, together with the corresponding database identifiers and overlapping genes. Because the input genes constituted a predefined targeted panel, these results were interpreted exclusively as functional annotation of the panel and not as independent or discovery-level pathway enrichment.

Supplementary Table S1a. Gene Ontology Biological Process annotation of the predefined gene panel. Significantly overrepresented Gene Ontology Biological Process terms identified for the predefined 17-gene panel. Terms are ordered according to the Benjamini–Hochberg-adjusted p-value. These results were interpreted as functional annotation of the predefined panel rather than as independent pathway discovery.

GO Biological Process term	GO ID	Overlapping genes	Adjusted p-value
Extracellular matrix organization	GO:0030198	ADAMTS5; MMP2; MMP9; MMP13; COL2A1; SPARC	1.2×10^{-6}
Collagen catabolic process	GO:0030574	MMP2; MMP9; MMP13; COL2A1	3.4×10^{-5}
Inflammatory response	GO:0006954	IL1B; IL1R1; IL1RAP; HP	2.1×10^{-4}
Cytokine-mediated signaling pathway	GO:0019221	IL1B; IL1R1; IL1RAP; JAK2; TYK2	4.8×10^{-4}
Response to wounding	GO:0009611	MMP2; MMP9; MMP13; SPARC; VEGFA	7.9×10^{-4}
Angiogenesis	GO:0001525	VEGFA; SPARC; IGFBP6	0.0021
Sensory perception of pain	GO:0019233	ASIC3; NGFB; NTRK1	0.0034

Supplementary Table S1b. WikiPathways annotation of the predefined gene panel. Significantly overrepresented WikiPathways 2024 Human terms identified for the predefined 17-gene panel. Terms are ordered according to the Benjamini–Hochberg-adjusted p-value.

WikiPathways term	WikiPathways ID	Overlapping genes	Adjusted p-value
OA chondrocyte hypertrophy	WP5373	ADAMTS5; MMP13; COL2A1	8.7×10^{-5}
Endochondral ossification	WP474	COL2A1; MMP13; SPARC	1.3×10^{-4}
Endochondral ossification with skeletal dysplasias	WP4808	COL2A1; MMP13	2.9×10^{-4}
IL-26 signaling	WP5347	IL1B; IL1R1; IL1RAP	0.0018
NF-κB survival signaling	WP3617	IL1B; IL1R1; IL1RAP; JAK2	0.0031

Supplementary Table S1c. KEGG annotation of the predefined gene panel. Significantly overrepresented KEGG pathways identified for the predefined 17-gene panel. Terms are ordered according to the Benjamini–Hochberg-adjusted p-value.

KEGG pathway	KEGG ID	Overlapping genes	Adjusted p-value
Inflammatory mediator regulation of TRP channels	hsa04750	IL1B; NGFB; NTRK1; ASIC3	6.1×10^{-5}
Th17 cell differentiation	hsa04659	IL1B; JAK2; TYK2	2.7×10^{-4}
Cytokine–cytokine receptor interaction	hsa04060	IL1B; IL1R1; IL1RAP; JAK2; TYK2	4.2×10^{-4}
MAPK signaling pathway	hsa04010	IL1B; NGFB; NTRK1	0.0019
PI3K–Akt signaling pathway	hsa04151	VEGFA; IGFBP6; SPARC	0.0038

Supplementary Table S1d. BioPlanet annotation of the predefined gene panel. Significantly overrepresented BioPlanet pathways identified for the predefined 17-gene panel. Terms are ordered according to the Benjamini–Hochberg-adjusted p-value.

BioPlanet pathway	BioPlanet ID	Overlapping genes	Adjusted p-value
Endochondral ossification	BP:000671	COL2A1; MMP13; SPARC	1.1×10^{-5}
Extracellular matrix organization	BP:000112	ADAMTS5; MMP2; MMP9; MMP13; COL2A1; SPARC	2.4×10^{-5}
Matrix metalloproteinase activity	BP:000298	MMP2; MMP9; MMP13	3.9×10^{-5}
Interleukin-mediated signaling	BP:000412	IL1B; IL1R1; IL1RAP	0.0012
Immune–stromal interactions	BP:000521	SPARC; HP; VEGFA; IGFBP6	0.0029
Leptin-mediated modulation of immune responses	BP:000733	IL1B; JAK2	0.0041
Oncostatin M signaling	BP:000812	IL1R1; IL1RAP; JAK2	0.0053

References

1. Slenter, D.N.; Kutmon, M.; Hanspers, K.; Riutta, A.; Windsor, J.; Nunes, N.; Mélius, J.; Cirillo, E.; Coort, S.L.; Digles, D.; et al. WikiPathways: A Multifaceted Pathway Database Bridging Metabolomics to Other Omics Research. *Nucleic Acids Res* **2018**, *46*, D661–D667, doi:10.1093/nar/gkx1064.
2. Hu, J.; Lepore, R.; Dobson, R.J.B.; Al-Chalabi, A.; M. Bean, D.; Iacoangeli, A. DGLinker: Flexible Knowledge-Graph Prediction of Disease–Gene Associations. *Nucleic Acids Research* **2021**, *49*, W153–W161, doi:10.1093/nar/gkab449.
3. Kuleshov, M.V.; Jones, M.R.; Rouillard, A.D.; Fernandez, N.F.; Duan, Q.; Wang, Z.; Koplev, S.; Jenkins, S.L.; Jagodnik, K.M.; Lachmann, A.; et al. Enrichr: A Comprehensive Gene Set Enrichment Analysis Web Server 2016 Update. *Nucleic Acids Res* **2016**, *44*, W90–W97, doi:10.1093/nar/gkw377.
